# Modeling the effect of linguistic predictability on speech intelligibility prediction

**DOI:** 10.1121/10.0017648

**Published:** 2023-03-17

**Authors:** Amin Edraki, Wai-Yip Chan, Daniel Fogerty, Jesper Jensen

**Affiliations:** 1 Department of Electrical and Computer Engineering, Queen's University, Kingston, Ontario K7L 3N6, Canada; 2 Department of Speech and Hearing Science, University of Illinois Urbana-Champaign, Champaign, Illinois 61820, USA; 3 Demant A/S, Smørum 2765, Denmark a.edraki@queensu.ca, chan@queensu.ca, dfogerty@illinois.edu, jesj@demant.com

## Abstract

Many existing speech intelligibility prediction (SIP) algorithms can only account for acoustic factors affecting speech intelligibility and cannot predict intelligibility across corpora with different linguistic predictability. To address this, a linguistic component was added to five existing SIP algorithms by estimating linguistic corpus predictability using a pre-trained language model. The results showed improved SIP performance in terms of correlation and prediction error over a mixture of four datasets, each with a different English open-set corpus.

## 1. Introduction

Speech intelligibility, defined as the degree to which an average listener correctly identifies a spoken message, is an important perceptual factor in adverse acoustic conditions and can be estimated in a listening test. The intelligibility scores obtained from a listening test generally depend on multiple factors that can be grouped into five categories:^1^ (1) acoustics (e.g., distortion severity), (2) listener characteristics (e.g., normal hearing vs hard-of-hearing listeners), (3) test material (e.g., speech corpus), (4) test equipment (e.g., sound reproduction equipment), and (5) listening test protocol/paradigm.

Speech intelligibility prediction (SIP) algorithms have primarily been designed to model the contribution of the first category: acoustics. As such, SIP algorithms can now account for the effects of a wide range of acoustic distortions and processing conditions on speech intelligibility.^2–6^ This ability to quickly and inexpensively predict speech intelligibility allows SIP algorithms to potentially replace expensive, time-consuming listening tests. However, while there has been some attention to certain listener characteristics, e.g., Refs. 3 and 7, which incorporate aspects of hearing deficiency, SIP algorithms generally ignore the other factors contributing to intelligibility. The restricted focus on acoustic factors limits the generalizability of SIP to other conditions, such as across different speech corpora.

The generalizability of SIP algorithms can be enhanced through consideration of the third factor category related to the redundancy or predictability of the test corpus that is known to affect listener intelligibility scores.^8^ For instance, changing the response vocabulary size has been shown to affect the listening test performance across different acoustic conditions.^9–11^ Such *linguistic* differences in the test corpus are not provided to many SIP algorithms, e.g., those operating at the acoustic signal level.^12–14^ Hence, such SIP algorithms would predict the same score for a given acoustic condition irrespective of the stimulus corpus, while the listening test scores may vary with the linguistic predictability of the corpus contents.

In the present study, we take a step toward improving SIP accuracy by incorporating linguistic predictability of the test corpus, as represented by context-dependent next-word probabilities estimated by a pre-trained language model. The estimated probabilities are used in a proposed probabilistic model to augment existing SIP algorithms. We evaluate the prediction performance of several state-of-the-art SIP algorithms with and without the linguistic component augmentation over several acoustic conditions with varying linguistic predictability. The results demonstrate the benefit of incorporating the linguistic component to improve SIP performance, particularly when considering intelligibility across different test materials. The present work is an initial feasibility study to demonstrate how language models can be combined with SIP to improve prediction performance.

This paper is structured as follows: Sec. 2 provides a definition of the problem and describes the scope of the work. Section 3 introduces information content as a proxy for linguistic predictability. The proposed linguistic augmentation is introduced in Sec. 4. Section 5 describes the intelligibility datasets. Implementation details are provided in Sec. 6. Experimental results are discussed in Sec. 7. Last, Sec. 8 concludes the work.

## 2. Problem definition

This section provides a practical definition of the problem and scope of the work. Existing SIP algorithms have commonly been evaluated using datasets with different acoustic conditions, but fixed linguistic characteristics (e.g., fixed test speech corpus). This approach evaluates the algorithms' ability to capture the effect of acoustic factors on speech intelligibility. However, the algorithms' performance in capturing the effect of linguistic predictability is not evaluated. In contrast to this traditional test setup, we evaluate the performance of the SIP algorithms across *different speech corpora* and different acoustic conditions. To this end, we merge multiple datasets, each with a different corpus and a range of acoustic conditions. The SIP algorithms are then evaluated on the union of the datasets. In this setup, the SIP algorithms under test are expected to capture the effect of (1) different acoustic distortions and (2) different speech corpora on speech intelligibility.

Five listening test datasets (one used for training, four used for testing) are examined that measured average open-set word recognition across a group of normal-hearing listeners for different corpora of meaningful English sentences. Each dataset is composed of: (1) an ensemble of degraded speech signals that represent realizations of each tested acoustic condition (along with a clean version for reference-based SIP), (2) word recognition scores averaged across listener participants and sentences for each acoustic condition, and (3) the text transcript of the listening test (i.e., corpus). To promote broad applicability, the proposed model augmentation is constructed using as little information from the listening test as possible. Therefore, while we assume that the text corpus is known, the exact subset of the corpus used to evaluate intelligibility in each acoustic condition is unknown. In the present work, we only consider macroscopic SIP algorithms^15,16^ that predict the average intelligibility of an acoustic condition and do not inherently model corpus predictability (such as SIP using automatic speech recognition^17–19^).

## 3. Information content as a proxy for linguistic predictability

In this section, we synopsize a computationally feasible proxy for linguistic predictability that can be used to augment existing SIP algorithms. In this regard, a large body of previous work suggests that humans are expectation-based language processors.^20–23^ For example, a language model based on transformers can be used as an effective substitute for the Cloze deletion test.^24^ In a Cloze test, participants are asked to fill in the blanks in a given text passage. We hypothesize that the next-word probabilities from a pre-trained language model can be used to model the effect of linguistic predictability on speech intelligibility. Using a pre-trained causal language model, the information content of a word *w_i_* (or word surprisal) in a sequence $\left(\ldots ,w_{i-2},w_{i-1},w_{i}\right)$ is defined as the conditional negative log-likelihood of the word:

$$e(w_{i})=-\text{log}\;P_{\theta }(w_{i}|w_{<i}),$$ (1)

where $e(w_{i})$ denotes the information content of the word *w_i_* given the context $\left(w_{<i}\right)$,^25^ *θ* denotes the parameters of the language model, and $P_{\theta }(w_{i}|w_{<i})$ is the probability assigned by the language model to the word *w_i_* given the context $\left(w_{<i}\right)$. For simplicity, we consider the causal setting where a word *w_i_* is conditioned on the past words $w_{<i}$.

## 4. Model description

This section describes the proposed model. In Sec. 4.1, we describe a general probabilistic approach to model the effect of *both* acoustic distortion and linguistic predictability on speech intelligibility of individual words. In Sec. 4.2, we use the concepts defined in Sec. 4.1 to develop a practical model that estimates the *average* intelligibility of an acoustic condition based on the assumptions and restrictions stated in Sec. 2. Finally, we present the model training procedure in Sec. 4.3.

### 4.1 A probabilistic approach to SIP

Consider a listening test in which participants listen to and try to identify a sequence of words. Let *C* denote the set of sentences forming the listening test corpus. Let *X* denote a binary random variable whose values indicate whether an average listener recognizes a word *w* (*X* = 1) or not (*X* = 0). Let *E* and *D* denote random variables whose values are the information content of the word and the corresponding SIP algorithm output, respectively. The computed/observed values of *D*, *E*, and *X* over a dataset form an ensemble of realizations of these random variables. We consider the output of the existing SIP algorithm D to be a proxy for severity of acoustic distortion and information content *E* to be a proxy for linguistic predictability. P(X=1|D,E) denotes the probability of a word being recognized in a listening test by an average listener, given its linguistic information content *E* and the output *D* of an existing SIP algorithm, and captures the effect of linguistic predictability of the corpus and severity of the acoustic distortion on word-level speech intelligibility.

### 4.2 Macroscopic SIP in practice

This section aims to develop a practical algorithm that estimates the average intelligibility of an acoustic condition. Assuming for now that P(X=1|D,E) is known [the procedure to estimate P(X=1|D,E) using a small training dataset will be presented in Sec. 4.3], the average listening test score s(α,C) of a given acoustic condition *α* can be estimated as

$$\hat{s}(\alpha ,C)=E_{E}\left[E_{D}\left[P\left(X=1|D,E\right)\right]\right],$$ (2)

where $\hat{s}(\cdot )$ is an estimate of s(·) and the right-hand side expectations are calculated over the distribution of SIP algorithm output *D* and the information content *E* over the subset of the corpus that was used to evaluate the intelligibility of the acoustic condition *α*.

Under the assumptions stated in Sec. 2, two practical issues must be addressed to calculate the expectations in Eq. (2). First, *D* is a random variable whose values are per-word SIP algorithm outputs. However, since only macroscopic SIP algorithms are considered, word-level SIP algorithm outputs are not available. To evaluate Eq. (2) even so, we assume that Eq. (2) can be simplified as follows:

$$\hat{s}(\alpha ,C)\approx E_{E}\left[P\left(X=1|D=E_{D}[D],E\right)\right]=E_{E}\left[P\left(X=1|D=d(\alpha ),E\right)\right],$$ (3)

where d(α) is the average SIP algorithm output for the acoustic condition *α*. This simplification implies that it is possible to estimate the average listening test score of a given acoustic condition, using only the average SIP algorithm output, effectively reducing the probability distribution of *D* for a fixed acoustic condition to its mean.

Second, the exact subset of the corpus used to evaluate the intelligibility of each acoustic condition is not provided. However, in a typical subjective listening test, a random subset of the corpus with enough sentences to reliably evaluate the speech intelligibility is used for each acoustic condition. In this case, it is reasonable to assume that the distribution of information content is identical over the subset used and the entire corpus. Hence, we can use the following sample mean as an estimate of the expected value in Eq. (3):

$$\hat{s}(\alpha ,C)\approx \frac{1}{\left|C^{*}\right|}\sum_{w\in C^{*}}P(X=1|D=d(\alpha ),E=e(w)),$$ (4)

where $C^{*}\subseteq C$ is a “large” random subset of the listening test corpus *C*, *e*(*w*) is the information content of the word *w*, and |·| denotes the cardinality of the set. The larger is the selected subset $C^{*}$, the better the estimate $\hat{s}(\alpha ,C)$ in Eq. (4) approximates the expected value in Eq. (3). Equation (4) defines the output of the proposed SIP method, which uses the output *D* = *d* of an existing SIP algorithm and information content *E* = *e* to estimate the average listening test intelligibility score of a given acoustic condition, thus incorporating a linguistic component into SIP.

### 4.3 The probabilistic intelligibility model

To demonstrate the proposed method, we employ a Bayesian approach to estimate P(X=1|D,E) used in Eq. (4):

$$P(X=1|D,E)=\frac{P(D,E|X=1)\;P(X=1)}{P(D,E)}.$$ (5)

For simplicity and mathematical tractability, a two-dimensional Gaussian distribution is assumed for P(D,E|X=1) and P(D,E|X=0), and a small training dataset is used to find the parameters of the Gaussian distributions. To estimate the parameters of P(D,E|X), the training dataset should report word recognition results and SIP algorithm output for individual words in the listening test corpus. However, as outlined in Sec. 2, such detailed information may not be available from existing datasets. To circumvent this issue, we use the information available to prepare the required data, as follows. Let $D_{\text{train}}$ and $C_{\text{train}}$ denote the training dataset with *N* acoustic conditions and its associated corpus, respectively. Also, let $C_{\text{train}}^{*}\subseteq C_{\text{train}}$ denote a large random subset of the corpus. First, we assume that $C_{\text{train}}^{*}$ was used to evaluate the intelligibility of all the acoustic conditions in the dataset. Next, to create the word recognition labels for the words in $C_{\text{train}}^{*}$, we use information content $-\text{log}\;P_{\theta }(w_{i}|w_{<i}),$ to create a rank list: for each acoustic condition *α* and its associated average listening test score *s*, we *assume* that the $s\times |C_{\text{train}}^{*}|$ words with the lowest information content (i.e., the words that are most easily predicted from their content) are recognized by the average listener and are labeled with *X* = 1. These words are treated as resulting in the average listening test score of $s=\frac{s\times |C_{\text{train}}^{*}|}{\left|C_{\text{train}}^{*}\right|}$. The rest of the words are labeled with *X* = 0.

The above approach is motivated by the hypothesis that in any specific acoustic condition, an average normal-hearing listener is *more likely* to identify the words with lower *information content*. We rely on SIP performance to validate the practicality of this assumption and do not claim that the words labeled with *X* = 1 were, in fact, recognized in the listening test. The average SIP algorithm output d(α) is used for all the words in each acoustic condition. The procedure is repeated for all *N* acoustic conditions in the training dataset, resulting in $N\times |C_{\text{train}}^{*}|$ training samples.

The parameters of the distributions P(D,E|X=0) and P(D,E|X=1) are calculated using maximum likelihood estimation. The prior distribution of word recognition *P*(*X*) is estimated as the class relative ( X=0 or X=1) frequencies in the training dataset. Once P(D,E|X=0), P(D,E|X=1), P(X=0), and P(X=1) are estimated, the joint probability distribution of *D* and *E* can be calculated as P(D,E)=P(D,E|X=0)P(X=0)+P(D,E|X=1)P(X=1).

## 5. Datasets

Five intelligibility datasets with roughly equal number of acoustic conditions (11–15 conditions) were considered in this study. The IEEE dataset introduced below was used for model training, while the remaining four datasets were used for testing. To minimize the differences in factors, such as listening test equipment and procedure, all the datasets were selected from a single research group.^27–29^

***IEEE dataset***: In Ref. 27, acoustic realization of Institute of Electrical and Electronics Engineers (IEEE)/Harvard sentences^30^ were corrupted by four types of noise: steady-state speech-shaped noise (SSN) and three types of time-compressed or expanded speech-modulated SSN. The noise modulation was time-compressed or expanded using pitch-synchronous overlap-add to run at 25%, 100%, or 400% of the original duration. Noises were presented at three SNR levels: −8, −4, and 0 dB. This resulted in a total of 4 (noise types) x 3 (SNRs) = 12 degradation conditions. Stimuli were presented to five normal-hearing listeners. For this dataset, intelligibility scoring was done using only the *keywords* in the sentence and information content was only calculated for these target keywords. An example sentence from the IEEE corpus with the keywords highlighted: “The birch canoe slid on the smooth planks.” The corpus consists of 720 phonetically balanced sentences, divided into lists of 10 each.

***HINT dataset***: In Ref. 28, acoustic realization of Hearing in Noise Test (HINT)^26^ sentences were convolved with room impulse responses simulated using the image method. The sentences were presented according to four reverberation times: T60 = 0.9, 1.2, 1.5, and 2.1 s, and three Direct-to-Reverberant Ratios (DRR): DRR = 0, −10, −20 dB. This resulted in a total of 4 (T60) × 3 (DRR) = 12 reverberation conditions. The stimuli were presented to 15 normal-hearing subjects. For this dataset, intelligibility scoring used all words in the sentence. An example sentence from the HINT corpus: “A boy fell from the window.” The corpus comprises 250 sentences divided into 25 lists.

***TIMIT dataset***: Acoustic realization of Texas Instruments Massachusetts Institute of Technology (TIMIT) sentences^31^ were bandpass filtered into 18 one-third octave bands. The Hilbert envelopes were extracted from each band and bandpass filtered. Two modulation bands were investigated: 0–8 Hz and 8–16 Hz. Filtered envelopes were combined with the original spectral components and summed across bands to re-synthesize the original speech sample with reduced temporal modulation cues. Contribution of the modulation depth of the sentences were assessed through amplitude compression/expansion of the consonant–vowel intensity ratio using the TIMIT phonetic markings. Two modulation bands, two manipulated segments (consonants/vowels), and three segment level settings (0.5×, 1×, 2×), plus three control conditions of the full sentence limited with temporal modulations filtered at 0–8, 8–16, or 0–16 Hz were tested. This resulted in 15 acoustic conditions tested in the presence of a 2 dB SNR signal-correlated noise. The stimuli were presented to 20 normal-hearing subjects. For this dataset, intelligibility scoring used all words in the sentence. An example sentence from the TIMIT corpus: “She had your dark suit in greasy wash water all year.” The TIMIT corpus is composed of 630 speakers each reading 10 phonetically rich sentences.

***SPIN datasets***: In Ref. 29, the revised Speech Perception in Noise (SPIN) sentences^32^ were used to assess the respective role of the acoustic temporal envelope and the temporal fine structure by adding noise to either component. The revised SPIN sentences include equal numbers of high and low predictability sentences, with strong and low contextual cues for the recognition of the target words, respectively. Eleven acoustic conditions were tested, and the stimuli were presented to 20 normal-hearing subjects. The listening test scores for the two groups of SPIN sentences (high vs low predictability) were considered separately, resulting in two datasets: SPIN-low and SPIN-high. For the SPIN datasets, the target word is the last word of every sentence and information content was only calculated for the target word. An example sentence from the SPIN-high corpus: “She made the bed with clean sheets”, and from the SPIN-low corpus: “The old man discussed the dive.” The SPIN corpus consists of 400 sentences divided into eight lists.

***MRG dataset***: The Merged (MRG) test dataset is formed by merging the datasets introduced in this section with different linguistic predictability. The IEEE dataset will be used in Sec. 6 for model training, as it covers the broadest range of listening test scores among the datasets. The rest of the datasets, i.e., HINT, TIMIT, SPIN-low, and SPIN-high, are merged to form the MRG dataset which will be used to evaluate the performance of the SIP algorithms.

## 6. Implementation and figures of merit

A pre-trained language model, OpenAI GPT,^33,34^ is used to calculate the next-word probabilities. This model has a maximum context length of *L* = 512 words. Note that all sentences are shorter than the maximum context length. A random subset of size $|C^{*}|=700$ words (70–110 sentences) is selected from each corpus to calculate information content. Five SIP algorithms are considered to evaluate the performance of the proposed method: the extended short-time objective intelligibility index (ESTOI),^2^ the hearing-aid speech perception index (HASPI),^3^ the weighted spectro-temporal modulation index (WSTMI),^35^ the coherence speech intelligibility index (CSII),^36^ and speech intelligibility in bits index (SIIB).^5^ The algorithms are selected based on their previously reported excellent performances in terms of correlation with listening test scores.^35^ The IEEE dataset is used for training, and the model is fixed for each SIP algorithm afterward.

Five figures of merit are used to evaluate the performance of the proposed approach: (I) the Pearson correlation coefficient, (II) the Spearman rank correlation coefficient, (III) the root-mean-squared error (RMSE), (IV) the concordance correlation coefficient (CCC), and (V) the Kendal's correlation coefficient (Kendall's *τ*) between the listening test scores and each SIP algorithm's output. The figures of merit are reported for three conditions: (I) no mapping (*No*), (II) a generic (*Gen*) mapping, and (III) the proposed linguistic mapping (*Ling*) applied to the output of each SIP algorithm. Note that the Spearman and Kendal's correlation coefficients, unlike the Pearson correlation coefficient and CCC, do not assume a linear correlation between the variables and may be more suitable for the current study.

In addition to *No* and *Ling* mappings, we also derive and apply a generic (*Gen*) corpus independent mapping to the output of the SIP algorithms. A sigmoid mapping of the form $\frac{1}{1+\text{exp}(ad+b)}$ is applied to the output *d* of the SIP algorithm.^12^ The parameters of the mapping, *a* and *b*, are calculated using least square fitting to the ground truth listening test scores of a fixed training (IEEE) dataset. The performance of the *Gen* mapping provides a baseline for the performance of SIP algorithms when the linguistic predictability of the corpus is not taken into account. As an alternative to the *No* and *Ling* mappings, SIP algorithm end-users have the option to use a generic mapping when ground truth listening test scores from the target application are not available.

## 7. Experiments and results

### 7.1 Information content

**Fig. 1.**
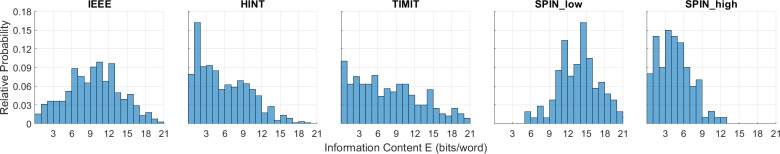
Binned normalized histograms of information content for different corpora.

Equation (1) is used with the pre-trained language model to estimate the information content for different corpora. Figure 1 shows the binned normalized histogram of information content for the corpora investigated in this study. The mean information content in bits per word for the corpora is as follows: SPIN-low (13.8), IEEE (9.6), TIMIT (7.9), HINT (5.8), and SPIN-high (4.5).

### 7.2 Posterior word recognition probability

**Fig. 2.**
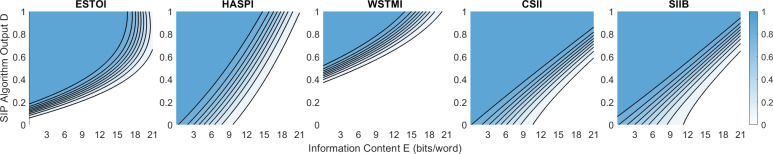
Posterior word recognition probability P(X=1|D=d,E=e) for different SIP algorithms. The IEEE dataset/corpus was used to estimate the posterior probabilities.

To visualize the estimated probability distribution P(X=1|D,E), Fig. 2 presents the posterior probability of a word being recognized in the training set, given its information content and the average SIP algorithm output. As is clear from Fig. 2, the words with lower information content, and acoustic conditions with higher average SIP algorithm output, are assigned a higher probability of being recognized by an average normal-hearing listener.

### 7.3 Performance evaluation

**Fig. 3.**
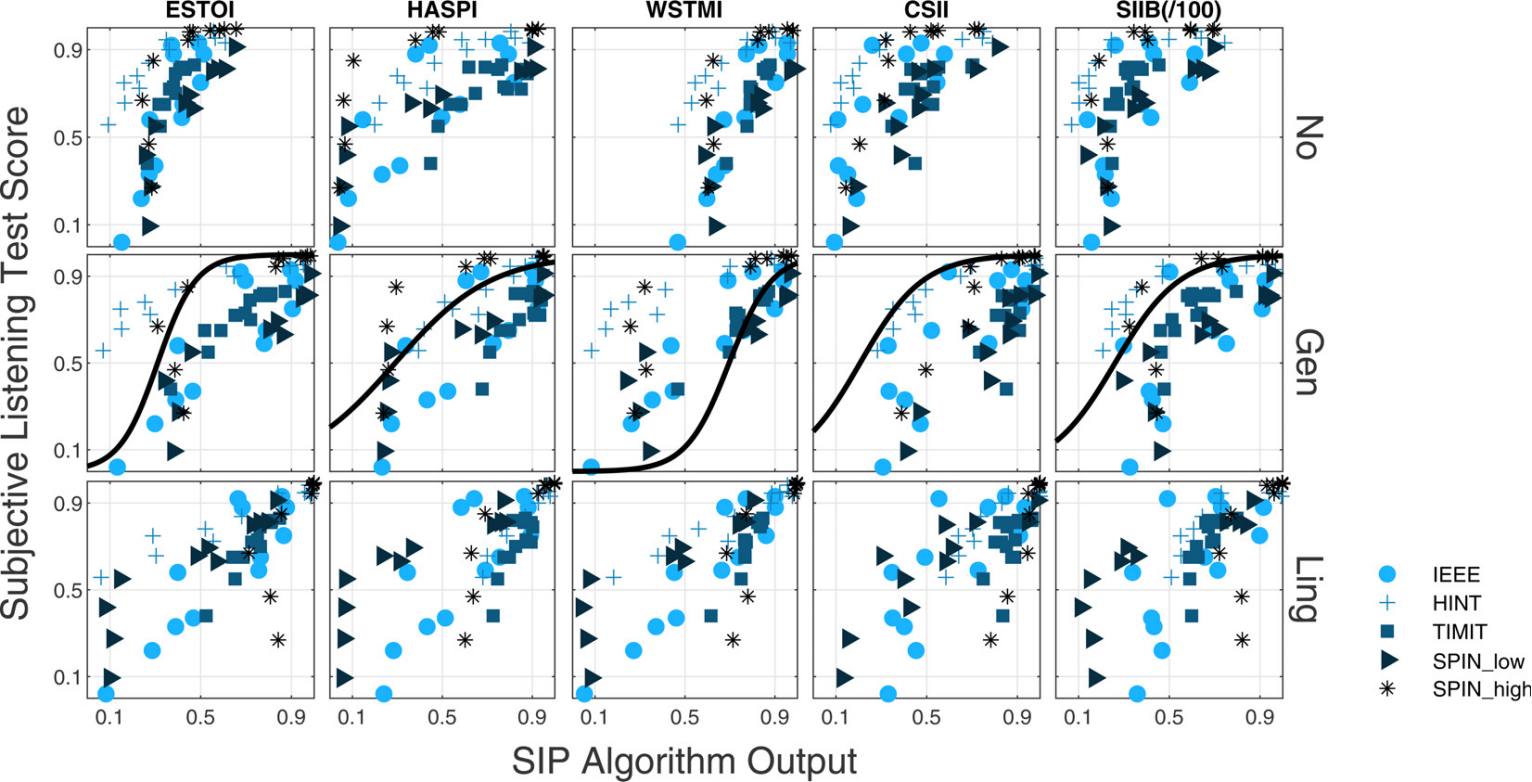
Scatterplots of the SIP algorithms output (horizontal axis) against listening test scores (vertical axis). Each column presents the results for one SIP algorithm, and each row presents the results for one mapping condition. The sigmoid mapping applied to the SIP algorithm outputs in the *Gen* mapping condition is also displayed for each SIP algorithm.

Figure 3 shows scatterplots of listening test and algorithm output scores for the datasets and SIP algorithm considered for the three mapping conditions described in Sec. 6 (*No*, *Gen*, and *Ling*). Each point in the scatterplots represents a different acoustic condition. The scatterplots qualitatively illustrate that the proposed linguistic mapping improves SIP performance across corpora with different linguistic predictability.

Table 1 shows the performance of the SIP algorithms considered in terms of the figures of merit on the MRG dataset. Each column presents the results for one performance metric and mapping condition, and each row presents the results for one SIP algorithm. The last row shows the mean performance across SIP algorithms. We only present the results for the MRG dataset, as it is the most relevant for this study and captures the SIP algorithms' performance across acoustic conditions with different linguistic predictability. (See supplementary material for the results for individual datasets).^1^ To determine statistically significant differences between the Pearson correlation values, pairwise comparisons using the Williams's t-test^37^ were performed for each algorithm between the mappings. The mapping that performed significantly better than both others (*p* < 0.05) is marked with (^*^) in Table 1.

**TABLE 1.** SIP performance on the MRG dataset. The MRG dataset was created by merging four intelligibility datasets, each with different acoustic conditions and a different corpus. The mapping that performed significantly better than the others (*p* < 0.05) in terms of the Pearson correlation coefficient is marked with (^*^).

Metric	Pearson			Spearman			RMSE			CCC			Kendall's *τ*		
Algorithm	No	Gen	Ling	No	Gen	Ling	No	Gen	Ling	No	Gen	Ling	No	Gen	Ling
ESTOI	0.63	0.64	0.75^*^	0.70	0.70	0.84	0.38	0.22	0.18	0.20	0.58	0.72	0.51	0.51	0.70
HASPI	0.69	0.71	0.77	0.66	0.66	0.85	0.29	0.18	0.17	0.51	0.69	0.75	0.48	0.48	0.67
WSTMI	0.65	0.65	0.78^*^	0.68	0.68	0.86	0.16	0.22	0.17	0.59	0.60	0.75	0.50	0.50	0.70
CSII	0.58	0.55	0.73^*^	0.58	0.58	0.79	0.36	0.20	0.16	0.27	0.55	0.71	0.42	0.42	0.62
SIIB	0.58	0.59	0.74^*^	0.65	0.65	0.81	0.43	0.24	0.17	0.20	0.49	0.71	0.46	0.46	0.65
MEAN	0.63	0.63	0.76	0.65	0.65	0.83	0.32	0.21	0.17	0.35	0.58	0.73	0.48	0.48	0.67

The results for the *No* mapping condition on the MRG dataset illustrate that the algorithms perform poorly when used across corpora with different linguistic predictability. Comparing the *No* and *Gen* columns in Table 1 shows that the *Gen* mapping improves the mean performance of the SIP algorithms in terms of RMSE and CCC over the *No* mapping condition by 34%, and 66%, respectively. This suggests that using a generic mapping with no knowledge of the listening test corpus may improve SIP performance across different corpora in terms of RMSE and CCC, but not Pearson, Spearman or Kendall's correlations. The performance of the generic mapping depends on the similarity between the data used to derive the mapping and the test dataset.

Comparing the results between the *Ling* and *Gen* mappings suggests that the linguistic component generally improves SIP performance in terms of all figures of merit when used across corpora with different linguistic predictability. With the linguistic mapping applied, the mean Pearson correlation coefficient, Spearman correlation coefficient, RMSE, CCC, and Kendall's *τ* across SIP algorithms improved by 21%, 28%, 19%, 26%, and 37% over the *Gen* mapping condition, respectively. Note that the linguistic mapping was derived separately from the generic mapping; with respect to *No* mapping, the improvements provided by the linguistic mapping are 21%, 28%, 47%, 109%, and 37% in terms of the mean Pearson correlation coefficient, Spearman correlation coefficient, RMSE, CCC, and Kendall's *τ* across SIP algorithms, respectively.

## 8. Future work and conclusion

Future work is required to investigate the following: (1) the effect of the performance of the language model (e.g., in terms of the perplexity score) on SIP performance, (2) the effect of using non-causal language models to calculate word probabilities, (3) the effect of incorporating “partially available context” (i.e., some words may be masked by noise/interference) to calculate the word probabilities, (4) using Cloze tests to derive the next-word probabilities, which also enables the model to be used across different languages and different test scenarios, (5) using microscopic SIP algorithms that exploit frame-level outputs to predict the intelligibility of individual words within sentences, (6) employing more sophisticated probability models and training procedures to further improve linguistic mapping performance beyond the generic mapping, and (7) the effect of talker characteristics, which was not considered here.

The results of this study suggest that SIP performance can be improved by accounting for corpus linguistic predictability. The *Gen* and *Ling* results together suggest that in general, applying a (monotonic) mapping to SIP algorithm outputs may improve SIP performance, particularly when used across corpora with different linguistic predictability, or when trying to predict the absolute speech intelligibility scores (as opposed to an index correlated with speech intelligibility). The mapping should be trained on subjective data as close to the target application as possible. Overall, the results demonstrate the feasibility and importance of including linguistic properties in SIP to improve generalizability across diverse test corpora that vary in linguistic predictability.

## Footnotes

^1^
See supplementary material at https://www.scitation.org/doi/suppl/10.1121/10.0017648 for the results for individual datasets
